# The Role of the Gastrointestinal Tract in Toxigenic *Clostridium tetani* Infection: A Case-Control Study

**DOI:** 10.4269/ajtmh.21-0146

**Published:** 2021-06-28

**Authors:** Nguyen Van Hao, Nguyen Ngoc My Huyen, Nguyen Thi Han Ny, Vo Thi Nhu Trang, Nguyen Van Minh Hoang, Duong Bich Thuy, Nguyen Thanh Nguyen, Pham Thi Lieu, Ha Thi Hai Duong, Tran Thi Diem Thuy, Phung Tran Huy Nhat, Dong Thi Hoai Tam, Maciej F. Boni, Lam Minh Yen, Le Van Tan, Tran Tan Thanh, James Campbell, C. Louise Thwaites

**Affiliations:** 1 University of Medicine and Pharmacy, Ho Chi Minh City, Vietnam;; 2 Hospital for Tropical Diseases, Ho Chi Minh City, Vietnam;; 3 Oxford University Clinical Research Unit, Hospital for Tropical Diseases, Ho Chi Minh City, Vietnam;; 4 Gia Dinh Hospital, Danang, Vietnam;; 5 Center for Infectious Disease Dynamics, Department of Biology, Pennsylvania State University, University Park, Pennsylvania;; 6 Centre for Tropical Medicine and Global Health, University of Oxford, Nuffield Department of Medicine Research Building, University of Oxford, Headington, Oxford, United Kingdom

## Abstract

Tetanus arises from wound contamination with *Clostridium tetani,* but approximately one fifth of patients have no discernable entry wound. *Clostridium tetani* is culturable from animal feces, suggesting the gastrointestinal tract could be an endogenous reservoir or direct-entry portal, but human data are lacking. In this study of 101 Vietnamese adults with tetanus and 29 hospitalized control subjects, admission stool samples were cultured for *C. tetani*. Anti-tetanus toxin antibodies were measured by ELISA. *Clostridium tetani* toxigenicity was evaluated using polymerase chain reaction and sequencing. Toxigenic *C. tetani* was cultured from stool samples in 50 of 100 (50%) tetanus cases and 12 of 28 (42.9%) control subjects (*P* = 0.50), and stool samples of 44 of 85 (52.4%) tetanus cases with clinically identified wounds compared with 6 of 15 (47.6%) patients without clinically identified wounds (*P* = 0.28). Nine of 12 (75%) control subjects with toxigenic *C. tetani* in their stool samples lacked protective antibody concentrations. These findings fail to show evidence of an association between gastrointestinal *C. tetani* and tetanus infection, but emphasize the importance of increasing vaccination coverage.

Tetanus remains a significant public health concern in many low- and middle-income countries. It is caused by the bacterium *Clostridium tetani*, an anaerobic bacterium able to persist in the environment as a resistant sporulated form. Not all strains of *C. tetani* cause disease, because toxigenicity is conferred by a plasmid containing the tetanus toxin gene.^1^ However toxigenic *C. tetani* strains have been cultured from many environments, including human and animal feces.^2–8^

Wounds contaminated with soil or manure are reported to be at high risk for tetanus acquisition, and management should be determined according to an assessment of exogenous contamination.^9^ Nevertheless, it is possible that gastrointestinal colonization with *C. tetani* represents an important route of endogenous contamination or direct portal of entry. Given the ubiquitous presence of *C. tetani* and the relative rareness of the disease, carriage has also been postulated controversially to cause “natural immunity” from tetanus.^10^

Studies of fecal *C. tetani* carriage in humans are limited to historical studies and yield conflicting results. Almost 100 years ago, Tenbroeck and Bauer^8^ isolated *C. tetani* capable of causing tetanus in mice in 27 of 78 stool samples from hospitalized patients in China, but Kerrin,^7^ working in the UK, failed to isolate any *C. tetani* from 300 human stool samples despite frequently isolating the toxigenic bacterium from a variety of animals using the same techniques.

In view of continuing uncertainties around the relationship between *C. tetani* carriage, disease, and immunity we carried out a case-control study in 101 adults with tetanus presenting to a tertiary referral hospital in Ho Chi Minh City, Vietnam.

The study was carried out at the Hospital for Tropical Diseases, a tertiary referral infectious disease center serving southern Vietnam (population, approximately 40 million). All adults older than 15 years admitted to the adult intensive care unit (ICU) at the Hospital for Tropical Diseases with generalized tetanus were eligible for admission to the study. Control subjects were patients admitted to the ICU with other diseases, likely to stay for more than 48 hours, and were matched for age and gender.

After enrollment, baseline characteristics and serum for antitoxin measurement were acquired for all patients. Tetanus cases received a careful examination for entry sites by a dedicated study doctor. This examination included search for oral and aural infection foci. Swabs for *C. tetani* culture were taken from any identified wound, as described previously.^11^ In all patients, the first stool sample after admission to the ICU was taken for *C. tetani* culture. Cultured *C. tetani* were tested for the tetanus toxin gene using polymerase chain reaction, as described previously.^11^ When relevant, Sanger sequencing of the polymerase chain reaction products was carried out to compare the sequences of toxin-coding genes obtained from the wound swab and the stool sample from the same patients. Tetanus antibody titers were measured by indirect ELISA, which was assayed in duplication using a tetanus toxoid (NIBSC: National Institute for Biological Standards and Control 04/150) and the anti-tetanus immunoglobulin standard 26/488. A cutoff of 0.1 IU/mL was taken as protective.^12,13^

Our sample size was based on our previous unpublished results of positive *C. tetani* stool culture rates of 75% in patients with tetanus and clinically identified entry sites, 90% with no identified entry site, and 45% in patients with central nervous system infections. We estimated sample size to detect two differences: 1) cases with known entry sites and control subjects, and 2) those with unknown entry sites and control subjects ( 80% power, two-sided 5% significance level, and with a case-to-control ratio of two). Our estimation was for 24 tetanus cases without entry sites and 12 control subjects, and 48 tetanus cases with known entry site and 24 control subjects.

Statistics were carried out using R v. 3.5.1 (R Foundation for Statistical Computing, Vienna, Austria). Median and interquartile range values are given for continuous data; number and percentage are provided for categorical data. Proportions were compared using the χ^2^ test. A *P* value of <0.05 was taken as significant. No corrections were made for multiple comparisons. The study was approved by the Ethical Committee of the Hospital for Tropical Diseases and the Oxford Tropical Research Ethics Committee. All participants, or their representatives, gave written informed consent prior to enrollment.

One hundred-one tetanus cases and 29 control subjects were enrolled between January and September 2018. Baseline demographics are shown in Table 1. Although age and gender were similar between cases and control subjects, control subjects were more likely to be residents of Ho Chi Minh City, admitted directly from the community and less likely to have manual-type occupations.

**Table 1:** Baseline characteristics of case and control populations

	Tetanus cases		Control subjects		
Characteristic	*N*	Count (%) or median (IQR)	*N*	Count (%) or median (IQR)	*P* value
Female	101	21 (20.8)	29	6 (20.7)	0.99
History of previous vaccination	101	2 (2.0)	29	1 (3.4)	0.649
Direct community admission	101	16 (15.8)	29	12 (41.4)	0.003
Resident of Ho Chi Minh City	16	16 (15.8)	13	13 (44.8)	0.002
Manual occupation: laborer, farmer, mechanic	55	42 (76.3)	27	10 (37.0)	< 0.001
Requiring mechanical ventilation	101	58 (57.4)	29	21 (72.4)	0.145
Survived to hospital discharge	101	95 (94.1)	28	18 (62.1)	< 0.001
Age (years)	101	52 (43–64)	29	53 (37–61)	0.483
Sequential Organ Failure Score	101	0 (0–1)	29	4 (3–8)	< 0.001
No. of comorbidities	101	0 (0–1)	29	1 (0–2)	< 0.001
ICU length of stay (days)	101	15 (8–23)	29	6 (3–10)	< 0.001
Hospital length of stay (days)	101	25 (18–35)	28	10.5 (8–17)	< 0.001
Diagnosis	Tetanus (*N* = 101)		Dengue (*N *= 4), encephalitis/meningitis (*N* = 2), encephalopathy (*N* = 2), pneumonia (*N* = 7), septic shock (*N* = 13), typhus (*N * = 1)		

ICU = intensive care unit; IQR = interquartile range.

Stool samples were available from 100 tetanus cases and 28 control subjects. Two participants (one tetanus patient and one control subject before stool samples were obtained). *Clostridium tetani* was cultured from stool specimens in 75 of 100 (75.0%) tetanus patients and 17 of 28 (60.7%) control subjects (*P* = 0.13). Fifty (50%) tetanus cases and 12 (42.9%) control subjects had bacteria that tested positive for the tetanus toxin gene (*P* = 0.50). There was no difference in the proportion of toxigenic *C. tetani* cultured from stool samples in those with known and unknown entry sites: 44 of 85 (52.4%) versus 6 of 15 (47.6%), respectively (*P* = 0.28).

Fifty-three patients with tetanus (53%) had identifiable wounds from which swabs could be taken. *Clostridium tetani* was cultured from 23 of these, and 19 were classified as toxigenic strains. Seven patients had toxigenic *C. tetani* isolated from both stool sample and wound swab. Of these, six had partial toxin-coding sequences recovered successfully from both wound swabs and stool samples. These were different in one to three amino acids (Table 2).

**Table 2:** Amino acid differences recorded in toxin coding gene of stool and wound samples of the same tetanus patients

		Location of toxin coding gene^*^		
Patient	Source	140	248	278
1	Stool	S	S	Q
	Wound	S	L	R
2	Stool	L	S	Q
	Wound	S	S	Q
3	Stool	S	L	NA
	Wound	L	S	Q
4	Stool	S	L	R
	Wound	L	S	Q
5	Stool	S	L	R
	Wound	S	S	Q
6	Stool	S	S	Q
	Wound	S	L	R

L = leucine; NA = not available; Q = glutamine; R = arginine; S = Serine; V = Valine.

* Codon position is relative to toxin gene of *Clostridium tetani* strain E88 (AF528097).

Antitoxin antibody titers greater than the protective threshold were measured in 7 of 29 (24.1%) control subjects and 9 of 101 tetanus cases (8.9%) (*P* = 0.03). Of these 9 tetanus cases, 8 were documented to have received antitoxin prior to blood sampling for antibodies, and one had been transferred from another hospital where prior antitoxin administration could not be excluded. Overall 9 of 12 (75%) control subjects with toxigenic *C. tetani* in their stool samples lacked protective antibodies. The median age of control subjects with protective antibodies was less than those without: 40 years (IQR, 35–44 years) and 57 years (IQR, 48–62 years), respectively (*P* = 0.047).

Our principal findings are that *C. tetani* was cultured frequently from study participants’ stool samples, and approximately half of the participants’ stool samples contained toxigenic strains, with a comparable detection rate between the cases and control subjects. High isolation rates occurred in patients with tetanus, who are often manual workers from rural locations, but we also observed high rates in control subjects. A potential limitation of our study is that control subjects, although matched for age and gender, were not matched for occupation. We found that control subjects were more likely to be Ho Chi Minh City residents with a lower likelihood of working in manual occupations. However, in view of our findings of high *C. tetani* isolation rates in control subjects, our study may indicate that *C. tetani* carriage is common in both rural and urban populations in Vietnam.

We found that 15% of tetanus cases had unknown entry sites. This number is less than previously reported (approximately 25%)^14^ and may be the result of the very careful search we made for these sites in our study. We did not find evidence supporting increased gastrointestinal isolation of *C. tetani* in patients with unknown entry sites, which suggests the gastrointestinal tract is not an important portal of entry for *C. tetani.* Because the majority of clinically identified entry sites in our study were small minor wounds or abrasions, it is possible that wounds in those with unknown entry sites may have healed before admission. Although most tetanus cases had toxigenic *C. tetani* isolated from stool samples, only a small number had toxigenic *C. tetani* isolated concurrently from clinically identified wounds. This may be the result of a failure to identify correctly the true entry portal or sampling after initial wound cleansing. The dissimilarities in genetic sequences between individuals’ isolates suggests wound and stool strains were often different; however, we were only able to obtain genetic sequencing in paired samples in a small number of patients.

It is possible that the *C. tetani* cultured from stool samples represents only the brief transit of ingested *C. tetani* spores through the gastrointestinal tract. Because we did not collect longitudinal stool samples, we cannot confirm whether persistent carriage was occurring. Literature reports have noted that *C. tetani* was cultured for only 4 days in stool samples from horses and mice after being fed *C. tetani* spores,^6,7^ but it was cultured for several months in Chinese hospital patients fed a “hospital diet.”^8^

The majority of the participants in our study lacked protective antibody concentrations of anti-tetanus toxoid antibody. Our use of ELISA for antibody measurement is a limitation of this study, because ELISA is subject to inaccuracy and specificity issues, especially at low antibody concentrations. As a result we used the recommended greater “protective threshold” for ELISA of 0.1 IU/mL, as opposed to 0.01 IU/mL recommended for toxin neutralization assay.^10^ Using this cutoff, only 24% of control subjects were found to have protective antibody concentrations, which is consistent with results of a previous sero-surveillance study from Ho Chi Minh City in which approximately 25% of males in this age group had protective antibody levels.^12^ Our findings also correspond to current vaccine policy in Vietnam, where immunization programs for infants and women of childbearing age have achieved high coverage rates; however, there are no programs for men and for adults born before immunization programs commenced, and there is limited access to later childhood tetanus–diphtheria boosters in many places.^15^ We note that nine cases of tetanus had antibody levels classified as “protective,” but a retrospective review of these cases revealed that eight had antitoxin documented prior to sampling for antibody measurement and the remaining case had been transferred from another hospital, where preceding antitoxin administration could not be excluded definitively.

We demonstrated the presence of toxigenic *C. tetani* in stool samples from a significant number of patients hospitalized for tetanus or other diseases. The majority of patients also lacked protective antibody levels against tetanus. Given the high frequency of toxigenic *C. tetani* present in stool samples, our findings emphasize that vaccination should remain the major strategy in tetanus prevention.
